# Only extraordinary volcanism can explain the presence of parts per billion phosphine on Venus

**DOI:** 10.1073/pnas.2121702119

**Published:** 2022-02-07

**Authors:** William Bains, Oliver Shorttle, Sukrit Ranjan, Paul B. Rimmer, Janusz J. Petkowski, Jane S. Greaves, Sara Seager

**Affiliations:** ^a^Earth, Atmospheric and Planetary Sciences, Massachusetts Institute of Technology, Cambridge, MA 02139;; ^b^School of Physics & Astronomy, Cardiff University, Cardiff CF24 3AA, United Kingdom;; ^c^Department of Earth Sciences, University of Cambridge, Cambridge CB2 3EQ, United Kingdom;; ^d^Institute of Astronomy, University of Cambridge, Cambridge CB3 0HA, United Kingdom;; ^e^Department of Astronomy & Astrophysics, Northwestern University, Evanston, IL 60201;; ^f^Center for Interdisciplinary Exploration and Research in Astrophysics, Northwestern University, Evanston, IL 60201;; ^g^Blue Marble Space Institute of Science, Seattle, WA 98154;; ^h^Cavendish Laboratory, University of Cambridge, Cambridge CB3 0HE, United Kingdom;; ^i^Department of Physics, Massachusetts Institute of Technology, Cambridge, MA 02139;; ^j^Department of Aeronautics and Astronautics, Massachusetts Institute of Technology, Cambridge, MA 02139

Truong and Lunine (1), in PNAS, recently described a model of the production of phosphine on Venus, in which phosphine is proposed to be the result of interaction of deep-mantle phosphides with acid in the clouds. We believe that it has two quantitative flaws.

The first is that erupted material will not reflect the chemistry of its deep-mantle source. Oxygen fugacity is a function of temperature, pressure, and rock composition (e.g., refs. 2 and 3). The oxygen fugacity of a rock of fixed bulk composition at the base of the lithosphere will be substantially different from the oxygen fugacity of the same rock in the deep mantle, even though they have the same intrinsic oxidizing capacity. Therefore, for the oxygen fugacity of the deep mantle to dictate the composition of volcanism at the surface (low pressure), the relaxation of a system to low-pressure thermochemical equilibrium (and its associated higher f(O_2_)) must be suppressed. Suppression implies rapid quenching of mantle material (on a timescale of minutes to hours), which is incompatible with the rate at which plumes raise deep-mantle material to the surface [tens of meters per year (4)]. On Earth, the overwhelming majority of erupted magmas sample the bulk chemistry of the upper mantle and lithosphere. The lithosphere of Venus is almost certainly oxidized, but, even if it is highly reduced, thermodynamic calculation shows that <10^−5^ of the phosphorus at upper-mantle pressures and temperatures will be present as phosphide (Fig. 1).

**Fig. 1.**
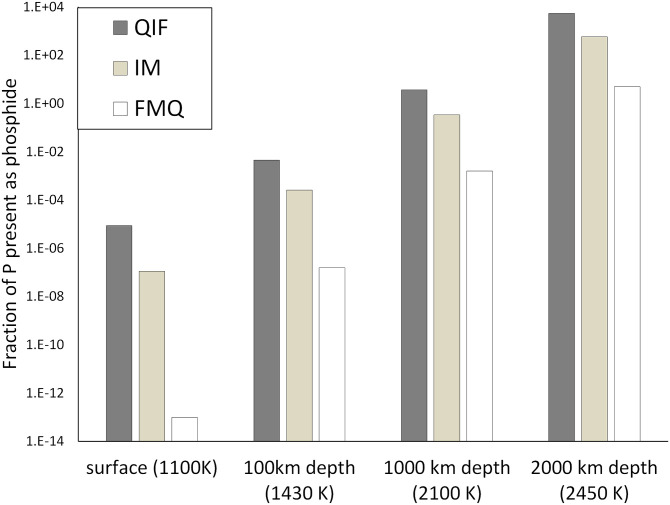
Under no plausible circumstances is more than 10^−5^ of phosphorus present as phosphide in erupted magmas; *y* axis: fraction of phosphorus present as phosphide (FeP) in silicates. Categories are depth (temperature) and oxygen fugacity (degree of reduction) of the erupta. Fe_3_P is three orders of magnitude less abundant. QIF, quartz/iron/fayalite buffer; IM, iron/magnetite buffer; FMQ, fayalite/magnetite/quartz buffer.

The second is that explosive volcanism is rare on Venus. Explosive volcanism requires rapid degassing of high loadings of volatiles in viscous melts. On Earth, such magmas are dominantly derived from either the interaction of intrinsically dry magma with surface water (producing phreatomagmatic eruptions) or the introduction of water and sulfur into the mantle source region of magmas by subduction. Neither subduction of hydrated oceanic crust nor magma–water interaction can happen on modern Venus, as it has no oceans. Reflecting this, the planet is degassing hydrogen-rich volatiles at 10% the rate of Earth (5). Rather, volcanism on Venus is expected to be effusive, and, consequently, far less efficient at converting magma to fine ash that could be brought to the cloud decks. This is confirmed by radar observations (6, 7). In addition, it is chemically inconsistent to postulate a water-rich rock that contains phosphide, as phosphide reacts with water at high temperatures.

These two factors alone require at least 21,600 km^3^/y of magma to deliver the required amount of phosphide to the clouds, not 0.03 km^3^/y as calculated by Truong and Lunine (1). This is roughly the equivalent of an Olympus Mons–scale volcano every 138 y. More realistic assumptions (e.g., regarding phosphine lifetime) require even more volcanism.

**Note Added in Proof**. A detailed, quantitative description of the model described here has been published recently (8).

## References

[1] N. Truong, J. I. Lunine, Volcanically extruded phosphides as an abiotic source of Venusian phosphine. Proc. Natl. Acad. Sci. U.S.A. 118, e2021689118 (2021). 34253608 10.1073/pnas.2021689118 PMC8307446

[2] B. R. Frost, “Introduction to oxygen fugacity and its petrologic significance” in Oxide Minerals: Petrologic and Magnetic Significance, D. H. Lindsleys, S. K. Banerjee, Eds. (Reviews in Mineralogy, Mineralogical Society of America, Washington, DC, 1991), vol. 25, pp. 1–10.

[3] E. M. Stolper, O. Shorttle, P. M. Antoshechkina, P. D. Asimow, The effects of solid-solid phase equilibria on the oxygen fugacity of the upper mantle. Am. Mineral. 105, 1445–1471 (2020).

[4] N. H. Sleep, Hotspot volcanism and mantle plumes. Annu. Rev. Earth Planet. Sci. 20, 19–43 (1992).

[5] F. Tian, E. Chassefière, F. Leblanc, D. A. Brain, “Atmosphere escape and climate evolution of terrestrial planets” in Comparative Climatology of Terrestrial Planets, S. J. Mackwell, A. A. Simon-Miller, J. W. Harder, M. A. Bullock, Eds. (University of Arizona Press, Tucson, AZ, 2013), pp. 567–581.

[6] P. K. Byrne, A comparison of inner Solar System volcanism. Nat. Astron. 4, 321–327 (2020).

[7] J. W. Head, L. Crumpler, J. C. Aubele, J. E. Guest, R. S. Saunders, Venus volcanism: Classification of volcanic features and structures, associations, and global distribution from Magellan data. J. Geophys. Res. Planets 97, 13153–13197 (1992).

[8] W. Bains et al, Constraints on the production of phosphine by Venusian volcanoes. Universe 8, 54 (2022).

